# Spontaneous nanosized liposome formation from crude dried lecithin upon addition of glycerol

**DOI:** 10.1038/s41598-024-80970-6

**Published:** 2024-12-02

**Authors:** Alejandro G. Marangoni, Erica Pensini

**Affiliations:** 1 Department Food Science, University of Guelph, Guelph, ON N1G2W1 Canada; 2 School of Engineering, University of Guelph, Guelph, ON N1G2W1 Canada

**Keywords:** Liposomes, Spontaneous, Glycerol, Critical packing parameter, Lecithin, Nanosized, Stress birefringence, Chemical physics, Membrane structure and assembly

## Abstract

Nanosized liposomal vesicles (NLV) were successfully prepared using natural sunflower lecithin without the use of high-pressure homogenization or filtration. Upon glycerol addition to dispersions of lecithin multilamellar vesicles (MLVs), these broke down spontaneously to liposomes with diameters in the range of 100–200 nm. Static light scattering demonstrated that glycerol addition above 30% (w/w) induced the complete transformation of MLVs into NLVs. Langmuir trough compression experiments showed a two-region compressional behavior. Upon 62% (w/w) glycerol addition, the compressional modulus of the liposomes decreased from 18.5 to 8.13 mN/m. Water activity and pulse NMR measurements also showed a divergence in behavior above 30% (w/w) glycerol. Liposomes were not birefringent in water but became strongly birefringent at and above 30% (w/w) glycerol, as determined by polarized light microscopy, and lost all birefringence above 80% (w/w). This was interpreted as the induction of stress-birefringence in the phospholipid bilayers above 30% (w/w) glycerol, and a relaxation of such stress above 80% (w/w) glycerol. We hypothesize that the mixture of phospholipids in the lecithin results in an effective non-zero intrinsic curvature for the molecular mixture, which lowers the bending energy of the bilayer, allowing for an easier break-up upon mixing. Secondly, glycerol addition decreases attractive van der Waals’ interaction between lamellae in an MLV, thus weakening the multilamellar liposome walls. Glycerol also affects bilayer stability by strengthening the hydrogen bond network of water, which will affect phospholipid headgroup hydration. All these factors result in the spontaneous breakdown of MLVs into NLVs.

## Introduction

Liposomes are important structures used in the encapsulation and delivery of drugs and bioactives. The large interest in liposomes arises due to their medical applications, mainly the intravenous delivery of drugs^1,2^. Since the discovery of “lecithin spherulites composed of concentric bilayers” by Alex Bangham in 1964^3^, the field has grown and matured enormously. Methods for the manufacture of liposomes with narrow size distributions were developed early^4^ and applied ever since^5–7^. The mechanisms responsible for the formation of liposomes were discussed by Lassic^8,9^. These methods included extrusion through polycarbonate filters of defined pore sizes^10,11^, high power ultrasonication, pH jump^12–14^, phospholipid film hydration surface^15^, French press, microfluidization, solvent (ethanol) injection, detergent solubilization and removal, reverse phase evaporation, and a few others^5–7^. While liposomes can be prepared using various combinations of polar lipids, a significant portion of research has predominantly focused on phosphatidylcholine. Saturated versions of this phospholipid are particularly favored due to their resilience against oxidative degradation. It is worth noting, however, that natural systems do not exclusively consist of saturated phosphatidylcholine. Instead, biological membranes are complex mixtures, composed of phospholipids, glycolipids and proteins.

In the pursuit of creating ever smaller liposomes for intravenous medical applications and precise delivery to tumors or specific tissues, some researchers may have overlooked the possibility of spontaneous liposome formation. While studies in the late 1980s explored the mechanisms of liposome formation, they did not progress beyond a certain point. During this period, questions emerged about whether phospholipid vesicles could spontaneously form and whether liposomes could be considered thermodynamically stable, setting them apart from oil-in-water emulsions, which are kinetically stable but not thermodynamically stable.

As discussed by Lasic^16^, the only way to obtain thermodynamically stable phospholipid vesicles is via a spontaneous formation process, without large energy inputs such as those involved with homogenization, pressure filtration or ultrasonication. When considering the elastic energy of bending per unit area (*e*) of an incompressible two-dimensional fluid, a model for a lipid bilayer, there is a term within the expression that corresponds to the natural curvature of the phospholipid(s), *C*_*0*_,

1 $$e = \frac{1}{2}K_{C} (C_{1} + C_{2} - C_{0} )^{2} + K_{G} C_{1} C_{2}$$

where *K*_*C*_ is the elastic modulus of curvature, *C*_*1*_ and *C*_*2*_ correspond to the two principal curvatures of a vesicle, and *K*_*G*_ is the elastic modulus of Gaussian curvature.

For most lipids and practically all single-component bilayers, this spontaneous curvature term, *C*_*0*_, is zero. This means that these molecules do not intrinsically have a curvature. However, in this work, Lasic also mentioned that spontaneous formation of thermodynamically stable liposomes was possible if the spontaneous curvature term of the bilayer was non-zero. This can be achieved by having two or more components in the bilayer. Moreover, Safran and coworkers^17^ hypothesized that when two surfactants are mixed, a vesicular phase can be stabilized by the curvature energy, which means that the vesicles are stable with respect to a “flat” lamellar phase, with a lower free energy. The explanation for this effect is that in a non-ideal mixture of surfactants of different shapes, these can basically arrange themselves in such a way as to stabilize this curved interface. This process would depend on the intrinsic “shape” of the phospholipids and their relative arrangement within the bilayer.

These authors took this idea even further and proposed that mixtures of two surfactants can lead to spontaneous vesicle formation. They showed that the energetic stabilization of vesicles can take place via the curvature elastic energy^18^. They also showed that the vesicles have a Gaussian distribution about an average size determined by the effective spontaneous curvature. In more recent work, Vincenzo Guida showed more thoroughly and convincingly that the formation of vesicle is thermodynamically favored by negative values of the Gaussian rigidity and by the presence of a spontaneous curvature of the amphiphilic membrane^19^. Again, he eventually resorted to the use of Isrealachvili’s critical packing parameter for this purpose.

So, if Lasic and Safran are correct, a heterogeneous mixture of phospholipids with a varied fatty acid composition could in principle spontaneously form thermodynamically stable liposomes. We therefore decided to test this hypothesis using “impure” commercially available deoiled lecithin powders derived from soybeans and sunflower seeds. These lecithins are the extracted phospholipids from the natural biological membranes of plants.

Thus, these “impure” and multicomponent phospholipid and glycolipid mixtures may be strong candidates for the spontaneous formation of thermodynamically stable liposomes. On the other side, and often neglected, is solvent composition and its effects on the Critical Packing Parameter (CPP). It would be advantageous to affect the structure of the liquid solvent (usually water) directing the self-assembly of phospholipids. Possible solvent structure modifiers include sugars and polyols. Polyols, such as glycerol, are soluble in water at any concentration.

Here we will explore the use of impure phospholipid mixtures to induce the formation of thermodynamically-stable Nanosized Liposomal Vesicles (NLV) spontaneously. The practical advantage of the approach we use is that the lecithin is dispersed directly in water, without the need to create a thin film by evaporating the phospholipids from an organic solvent, and slow hydration thereafter. In previous work^20^, researchers managed to encapsulate bioactives directly into MLVs, which upon shearing in a rotostator, yielded ~ 100 nm liposomal vesicles loaded with hydrophobic bioactives, such as tetrahydrocannabinol^20^ and phytosterols^21^. These would not qualify as spontaneous liposomes, but were relatively simple to prepare.

## Materials and methods

### Phospholipids

The soybean lecithin used was Phospholipon20 (Lipoid GmbH, Ludwigshafen, Germany), while the sunflower lecithin used was Sunlec25 and Sunlec65 from Perimondo (New York, NY, USA). The number refers to the phosphatidylcholine content of the mixture. The phospholipid composition of these samples was provided by the manufacturers, while the fatty acid composition was determined by us.

### Liposome preparation

We simply dispersed both powders at a 10% (w/w) level in deionized water at 22 °C. No thin film hydration was required. The powder dispersions were gently stirred with an overhead paddle mixer at 200 rpm for 18 h (overnight) at 22 °C. All the powder was dissolved/dispersed after this. It is important to allow sufficient time for hydration of the phospholipids since there are fractions that take at least one hour to disperse. For samples with glycerol added, we followed two methods, dilution after liposome dispersion (disperse in water, then add glycerol), or initial dispersion in the final medium composition (glycerol + water). Both methods yielded the same results. Glycerol was purchased from Fisher Scientific (St. Louis, MO, USA).

### Fatty acid analysis

Fatty acid methyl esters were prepared using a transmethylation procedure developed by Christie^22^. We conducted fatty acid composition analysis using an Agilent 6890-series gas chromatograph (Agilent Technologies, Inc.; Wilmington, DE, USA) equipped with a 7683-series auto-sampler and a BPX70 (SGE Inc.; Austin, TX, USA) GC column (60 m × 0.22 mm internal diameter; 0.25 μm film thickness). The temperature of the oven was increased from 110 to 230 °C (4 °C /min) and maintained at 230 °C for 18 min. The injector was set to 250 °C, operating at 20.1 psi and a flow rate of 17.7 mL/min. The carrier gas was high-purity helium, flowing at a velocity of 25 cm/s. We useda flame ionization detector (255 °C; 450 mL/ min air flow; 50 mL/min helium flow). The GC peaks we obtained were analyzed with Open LAB software (Agilent Technologies). The composition of fatty acids was determined by comparing the retention times with those of internal standards.

### Static light scattering

Static light scattering measurements were conducted using a Mastersizer 2000 (Malvern Instruments Ltd., UK) with a Hydro 2000SM small volume sample dispersion unit, to determine particle size distributions. The refractive index of the suspended particles was assumed to be similar to that of phospholipids, and the continuous phase was deionized water. Refractive index values of 1.42 and 1.33 were used for the dispersed and continuous phases, respectively. Samples were added at an obscuration of ~ 15%. Measurements were done in triplicate, using at least three different samples. All measurements were carried out at room temperature (22 °C).

### Compression isotherms

Tests were performed at 22⁰C with a Kibron Microtrough G1 Langmuir–Blodgett trough (having an area of 16,500 mm^2^), controlled by KBN LayerXPro software. Dispersions of Sunlec25 lecithin liposomes in water or 62% glycerol in water (w/w) were poured in the trough and the films were equilibrated for 5 min prior to each measurement. The final liposome concentration in the trough was 0.01% w/v. Symmetric mobile barriers were then used to compress films at the air–water interface, at a constant speed of 20 mm/min. The interfacial pressure exerted on a Wilhelmy plate was constantly measured during compression.

Data were fitted to the model of Marczak and coworkers^23^. This model is used for the compression of particles layers and describes a near sigmoidal curve characterized by a surface pressure plateau at a minimum linear particle distance (MLPD). Here all the particles are in contact with each other. The area corresponding to 50% if this MLPD pressure plateau is denoted *A*_*L*_. The model has the following form,

2 $$\Pi = \sum\limits_{i = 1}^{n} {\frac{{\Pi_{MLPD,i} }}{{1 + (A/A_{L,i} )^{\varphi } }}}$$

The model was fitted to the excess surface pressure (surface pressure—surface pressure at infinite area) data by non-linear regression using GraphPad Prism 8.4.2 (GraphPad Software, San Diego, CA, USA), as described in Marshall and coworkers^24^. We imposed no constraints or weighting in the data analysis, which was fitted using a medium convergence criterium and 1000 iterations. This approach was sufficient for convergence. The model was fitted to the data using asymmetrical, profile-likelihood confidence intervals. These were markedly more robust than the typical symmetrical (asymptotic) approximate confidence intervals. Data were fitted to a single compression isotherm (i = 1) and two serial compression isotherms (i = 2) simultaneously. A two-species model was chosen only if it offered a statistically significant improvement (P < 0.05) in the fit to the data over a one-species model. Fits of the model to the data yielded a series of parameters, including the MLPD surface pressure ($\Pi_{MLPD}$, mN/m), the area corresponding to 50% of the $\Pi_{MLPD}$, *A*_*L*_, and the maximum compressibility modulus ($C_{S}^{ - 1}$, mN/m), which was calculated as,

3 $$C_{S}^{ - 1} = \frac{{\varphi \Pi_{MLPD} }}{4}$$

where $\varphi$ is the exponential term in Eq. (2). For a more complete understanding of these parameters, the reader is directed to Marczak and coworkers^23^.

### Small-deformation rheology

The shear dynamic moduli of 5% (w/w) Sunlec25 sunflower lecithin liposomes in water or glycerol-water mixtures were determined using a rotational rheometer (MRC 302, Anton Paar, Graz, Austria), equipped with a 17 mm by 43 mm concentric cylinder (CC17/T200/AL). The unit was temperature-controlled using an attached water bath, ensuring that the sample was at a constant temperature of 22 °C. The concentric cylinder was filled with suspensions up to its maximum volume. Dynamic oscillatory shear measurements were conducted at a frequency of 1 Hz. Shear strain sweeps (amplitude sweeps) were carried out from 0.01 to 1000% shear strain. Samples were analyzed in at least duplicate for two replicate determinations. The data was analyzed using RheoCompass Software (Anton Paar, Graz, Austria).

### Viscosity

The dynamic viscosity of 5% (w/w) Sunlec25 sunflower lecithin liposomes in glycerol-water mixtures was determined using a rotational rheometer (MRC 302, Anton Paar, Graz, Austria) equipped with a 17 mm by 43 mm concentric cylinder (CC17/T200/AL). The temperature was kept constant at 25 °C using an attached water bath. The concentric cylinder was filled up to its maximum volume with suspensions. The viscosity of the samples was determined as a function of shear strain rate from 1 to 100 s^−1^. All samples displayed Newtonian behavior. Samples were analyzed in at least duplicate for two replicate determinations. The data was analyzed using RheoCompass Software (Anton Paar, Graz, Austria).

### Water activity

The water activity of mixtures of glycerol and water were carried out using an Aqualab 4TE benchtop water activity meter (Aqualab Corporation, Pullman, WA, USA). Mixtures of glycerol and water contained varying mass fractions, from 0 to 100%, in 10% increments. We added 5% (w/w) of Sunlec25 sunflower lecithin to these solutions, mixed at room temperature in a beaker over a magnetic stir plate, and allowed to equilibrate overnight before measurement. The machine carries out measurements automatically at a set temperature of 22 °C.

### Low-field pulsed NMR spectrometry

Proton spin–spin relaxation times (T_2_ relaxation values) were obtained for the glycerol-water-lecithin mixtures. Sunlec 25 lecithin was hydrated in the different glycerol-water mixtures at 5% (w/w) concentrations. Measurements were carried out on a 20 MHz (0.47 T) mq 20 series bench-top NMR spectrometer (Bruker Corp., Milton, ON, Canada). The sample chamber was kept at room temperature (22 °C). The free induction decay was acquired from a Carr-Purcell-Meiboom-Gill (CPMG) spin echo pulse train^25,26^, using 32 scan repetitions. We optimized the 90° and 180° pulse lengths using an automated calibration procedure, with characteristic values of approximately 2.6 μs and 6.2 μs, respectively. The pulse delay τ was set to 150 μs. Samples were transferred into small, disposable glass NMR tubes (height: 40 mm; diameter: 7 mm). A free induction decay (FID) was collected at room temperature. T_2_ relaxation profiles were obtained by processing the FID data with the CONTIN algorithm (Bruker Corp.), which extracts multiple rate constants using an inverse Laplace transform. The peak relaxation times were extracted using the PeakFit software package (v4.12, Systat Software Inc., San Jose, CA, USA).

### Light microscopy

Liposomes microstructure was investigated using bright field microscopy using an Omax M838PL-C180U3 light microscope (OMAX; Kent, WA, USA). Sunlec25 liposome suspensions were placed on glass microscope slides and covered with a coverslip. Samples were imaged using an 18 MP digital camera and analyzed using ToupView software (v3.7, ToupTek Photonics; Hangzhou, China). For polarized light microscopy, the polarizer lenses were placed at full extinction (90°). We took images at two different light intensity settings, mid-range (setting 3/6) and high intensity (setting 6/6). We report here the mid-intensity results. High intensity results were equivalent, but sample images were more saturated.

### Cryogenic transmission electron microscopy

Cryogenic transmission electron microscopy (Cryo-TEM) was performed by transferring 5 μl of each Sunlec25 sample onto Quantifoil Multi hole grids, which had been glow discharged. The suspension was thinned by blotting with filter paper, then plunged into liquid ethane which had been held close to liquid nitrogen temperature. The grid was stored in liquid nitrogen prior to being loaded into a pre-cooled holder, which is inserted into the Tecnai TEM (Thermo Scientific, USA). Samples were viewed at -175 °C and 200 kV. Images were recorded using the Gatan 4 K camera and the Gatan Digital Micrograph software (Gatan Inc., Roper Technologies, USA).

### Calculation of critical packing parameter

The critical packing parameter (CPP) was defined by Israelachvilli^27^ as

CPP = Hydrophobic volume/(Hydrophobic length*area of the hydropobic/hydrophilic interface).

The calculation of the CPP for different phospholipid molecules were carried out using Molecular Modelling Pro Plus (MMP +) version 8.1.40 (Norgwyn Montgomery Software Inc, James A. Quinn, lead programmer). The different phospholipids studied were drawn in the software package to predict the CPP. The exact calculations are described in the attached Supplementary File.

## Results and discussion

The fatty acid composition is very similar between the two lecithins, except for the higher linolenic acid (18:3) content of soybean lecithin (Table 1). In terms of phospholipid composition, both sunflower and soybean have similar phosphatidylcholine contents, about 25% (w/w), while the phosphatidylethanolamine content of soybean lecithin is about 1.7-fold higher than that of sunflower lecithin (24% vs. 14%), while sunflower lecithin contains about 1.7 times more phosphatidylinositol than soybean lecithin (24% vs. 15%). Regardless, this is a very heterogenous mixture of phospholipids, reflecting the composition of the native cellular membranes in the oilseed. To understand the ability of specific phospholipids to spontaneously form small vesicles*, i.e.* liposomes, we calculated the critical packing parameter (CPP) of different phospholipids, with different polar heads, as well as of different fatty acid composition. Results of these computer simulations are shown in Table 2.

**Table 1:** Phospholipid and fatty acid composition of the lecithin used in this work (Sunlec25) and a soybean derived lecithin for comparison purposes.

Phospholipid	Sunlec25 sunflower (w/w %)	Phospholipon20 Soybean (w/w %)
Phosphatidylcholine	25	25
Phosphatidylinositol	24	15
Phosphatidylethanolamine	14	24
Phosphatidic acid	7	7
Minor phospholipids	5	6
Lysophosholipids	6	8
Glycolipids	18	15

Fatty acid	Weight %	Weight %
16:0	17.6	18.9
18:0	4.1	4.0
18:1	11.1	9.7
18:2	64.7	58.8
18:3	n.d	6.6

**Table 2:** Predicted critical packing parameter for different phospholipid types and fatty acid compositions.

Fatty acid at sn-1 and sn-2	Phospholipid	Critical packing parameter
	*Phosphatidylcholine*	
Linolenic–Linolenic		0.96
Linolenic–Linoleic		0.92
Linoleic–Linoleic		0.84
Oleic–Linoleic		0.85
Oleic–Oleic		0.84
Palmitic–Linoleic		0.79
Palmitic–Oleic		0.80
Palmitic–Palmitic		0.73
	*Phosphatidylethanolamine*	
Linolenic–Linolenic		1.10
Linolenic–Linoleic		1.10
Linoleic–Linoleic		1.00
Oleic–Linoleic		1.01
Oleic–Oleic		1.01
Palmitic–Linoleic		1.03
Palmitic–Oleic		1.01
Palmitic–Palmitic		1.03
	*Phosphatidylinositol*	
Linolenic–Linolenic		0.77
Linolenic–Linoleic		0.77
Linoleic–Linoleic		0.81
Oleic–Linoleic		0.80
Oleic–Oleic		0.80
Palmitic–Linoleic		0.68
Palmitic–Oleic		0.75
Palmitic–Palmitic		0.59
	*Phosphatidylserine*	Protonated|Ionized
Linolenic–Linolenic		1.14|1.00
Linolenic-Linoleic		1.14|0.97
Linoleic–Linoleic		1.16|0.96
Oleic–Linoleic		1.15|0.95
Oleic–Oleic		1.15|0.97
Palmitic–Linoleic		1.14|0.92
Palmitic–Oleic		1.14|0.98
Palmitic–Palmitic		1.10|0.97
	*Phosphatidic Acid*	
Linolenic–Linolenic		0.89
Linolenic–Linoleic		0.88
Linoleic–Linoleic		0.84
Oleic–Linoleic		0.84
Oleic–Oleic		0.84
Palmitic–Linoleic		0.88
Palmitic – Oleic		0.93
Palmitic–Palmitic		0.94
	*Phosphatidylglycerol*	
Linolenic–Linolenic		0.60
Linolenic-Linoleic		0.60
Linoleic–Linoleic		0.58
Oleic–Linoleic		0.58
Oleic–Oleic		0.60
Palmitic–Linoleic		0.56
Palmitic–Oleic		0.56
Palmitic–Palmitic		0.56
	*Lyso-Phosphatidylcholine*	
sn-2 Linolenic PC/sn-1 Linolenic PC		0.20/0.21
sn-2 Linoleic PC/sn-1 Linoleic PC		0.20/0.22
sn-2 Oleic PC/sn-1 Oleic PC		0.23/0.23
sn-2 Palmitic PC/sn-1 Palmitic PC		0.20/0.22
	*Lyso-Phosphatidylethanolamine*	
sn-2 Linolenic PE/sn-1 Linolenic PE		0.46/0.45
sn-2 Linoleic PE/sn-1 Linoleic PE		0.47/0.46
sn-2 Oleic PE/sn-1 Oleic PE		0.46/0.46
sn-2 Palmitic PE/sn-1 Palmitic PE		0.45/0.44
	*Lyso-Phosphatidylinositol*	
sn-2 Linolenic PI/sn-1 Linolenic PI		0.060/0.090
sn-2 Linoleic PI/sn-1 Linoleic PI		0.060/0.092
sn-2 Oleic PI/sn-1 Oleic PI		0.064/0.092
sn-2 Palmitic PI/sn-1 Palmitic PI		0.064/0.100
	*Lyso-Phosphatidylserine*	Protonated|Ionized
sn-2 Linolenic PS/sn-1 Linolenic PS		0.33/0.30|0.25/0.22
sn-2 Linoleic PS/sn-1 Linoleic PS		0.36/0.36|0.20/0.28
sn-2 Oleic PS/sn-1 Oleic PS		0.31/0.31|0.25/0.25
sn-2 Palmitic PS/sn-1 Palmitic PS		0.30/0.32|0.27/0.26
	*Lyso-Phosphatidic Acid*	
sn-2 Linolenic PA/sn-1 Linolenic PA		0.46/0.44
sn-2 Linoleic PA/sn-1 Linoleic PA		0.42/0.40
sn-2 Oleic PA/sn-1 Oleic PA		0.40/0.40
sn-2 Palmitic PA/sn-1 Palmitic PA		0.39/0.38

Vesicles form more readily if the phospholipids have CPPs between 0.5 and 0.85. These simulations suggest that phosphatidylinositol and phosphatidylglycerol are the most promising molecules for vesicle formation. However, more importantly, phosphatidylcholine can form vesicles more readily if it contains palmitic, oleic and linoleic fatty acids in different combinations. The CPP of phosphatidylcholine containing linolenic-linoleic and linoleic-linoleic combinations should be avoided since the CPP is close to 1, inducing the formation of planar bilayers. However, combinations of palmitic, oleic and linoleic fatty acids have CPPs of 0.85 and lower. All lysophospholipids, with very low CPPs, form micelles, while phosphatidylserine and phosphatidylethanolamine have CPPs above 1, thus favoring the formation of planar bilayers or reverse micelles. Interestingly, phosphatidic acid could also help form liposomal vesicle spontaneously since it has a CPP of ~ 0.8–0.9. These values of CPP tend to induce planar bilayer formation, as in the case of very large vesicles or cells. In what follows, we will use complex mixtures of the above listed phospholipids in the presence of glycerol and water to study their ability to form liposomes.

**Fig. 1:**
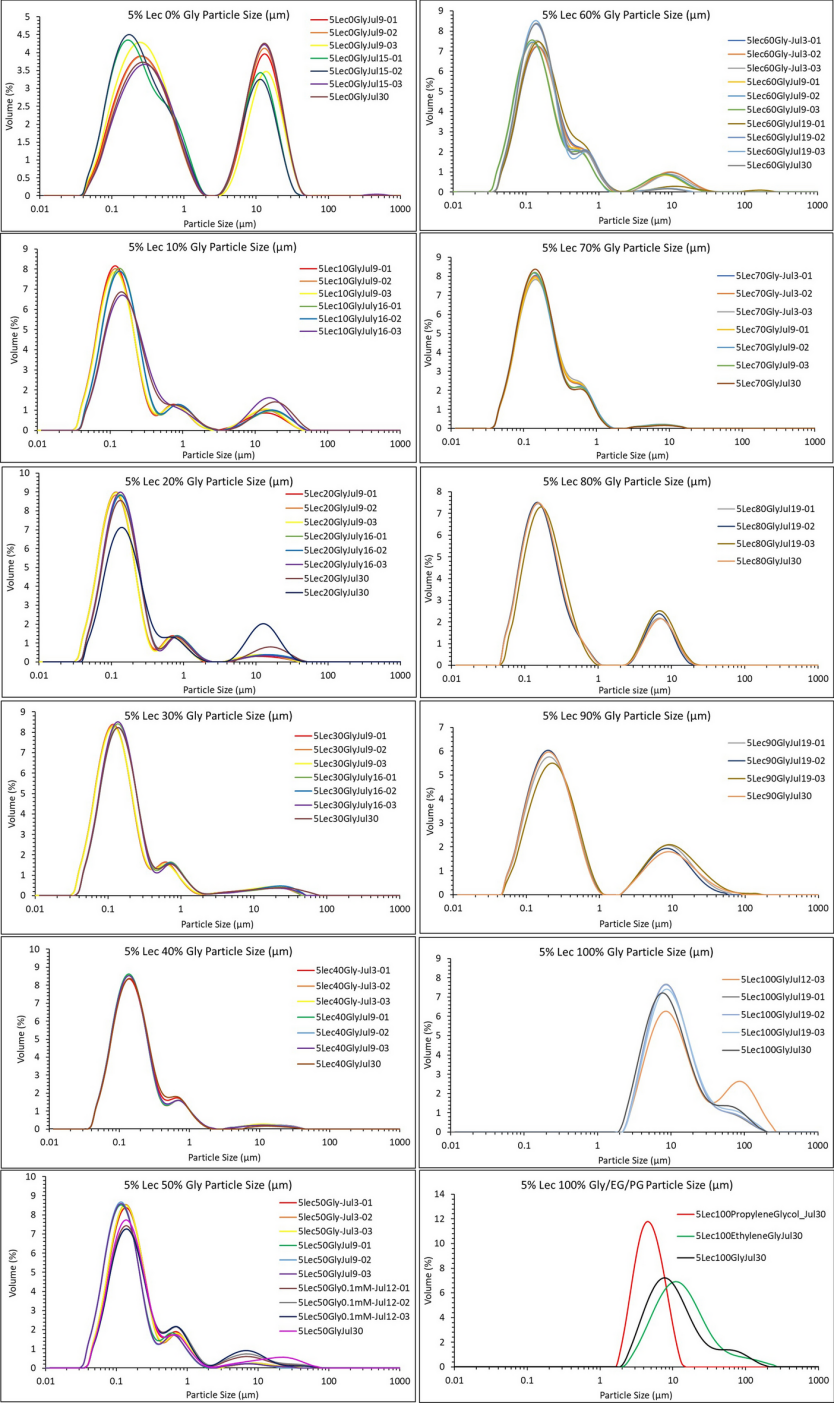
Particle size distributions for 5% (w/w) Sunlec 25 lecithin liposomes prepared in glycerol-water mixtures, from 0 to 100% (w/w) glycerol, in 10% (w/w) increments. The different profiles correspond to experimental replicates.

Figure 1 presents the main findings of this study. Sunflower lecithin (5% w/w) was dispersed in water in a beaker using a magnetic stirrer for an hour at room temperature (22 °C). Under these gentle conditions, a mixture of 10–15 µm multilamellar vesicles (MLV) and 150-250 nm vesicles was formed spontaneously (upper left-hand side graph). Note that light scattering reveals that aggregates are present even without water. However, without water, aggregates are not vesicles, as shown by brightfield and polarized light microscopy observations, which are discussed later.

**Fig. 2:**
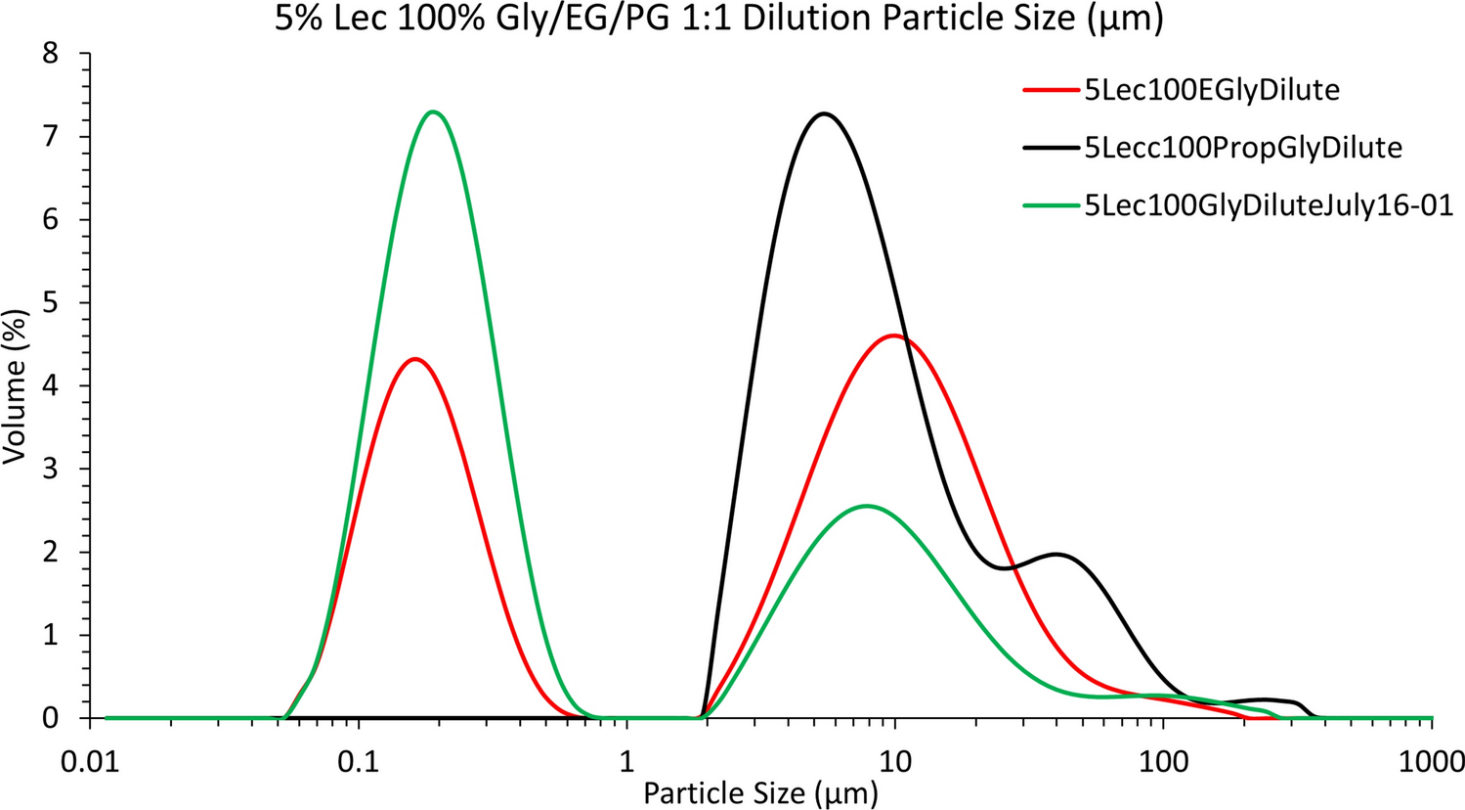
Particle size distributions for 5% (w/w) Sunlec 25 lecithin liposomes prepared in glycerol, propylene glycol and ethylene glycol and diluted 50% with water at room temperature without stirring. The different curves shown represent replicate experiments.

There was a visible polydispersity in the different replicate determinations, representative of the heterogenous size distribution of the vesicles present. Even after only 10% (w/w) addition of glycerol, however, the MLV population was drastically reduced, while the size distribution of smaller vesicles was much narrower and centered around 100 nm. Since we did not determine whether these liposomes are unilamellar (i.e., one phospholipid bilayer) or multilamellar (two or more phospholipid bilayers), we will refer to these as “nanosized liposomal vesicles” or NLVs, instead of SUVs (small unilamellar vesicles) or LUVs (large unilamellar vesicles). Upon 30% (w/w) glycerol addition, the MLV population almost completely disappeared, and remained as such up to 70% (w/w) glycerol. Interestingly, at 80% (w/w) and above, the MLV population returned, and for 100% glycerol, the NLVs could not be observed at all. These results are reminiscent of the study by Burni and coworkers^28^ (2024), where a similar effect was observed for water dispersions of soy lecithin upon addition of ethanol. Initially, unilamellar vesicles (ULV) of about 100 nm were formed above 10% (w/w) ethanol, but from 37 to 45% (w/w) ethanol, MLVs were formed again. We assume that below 10% (w/w) ethanol, the turbid lecithin dispersions were composed of MLVs, but the authors did not show this. The authors explained these effects based on the balance between the Edge (E_Edge_) and Bending (E_Bend_) energies of the phospholipid bilayers. There would be a high energetic cost for exposing the hydrophobic "edge” of a bilayer to an aqueous environment, and thus a closed vesicle formation is enhanced. As long as E_Bend_ <  < E_Edge_, a small unilamellar vesicle will form. As the solvent becomes more hydrophobic upon ethanol addition, the Edge energy drops and the bilayer sheets will not need to bend as much in the presence of ethanol and hence will form larger MLVs. This is exactly what we observed in our work, where NLVs were formed between 10 and 60% glycerol, and MLVs were observed at and above 70% glycerol (Fig. 2). We tried changing solvents to propylene glycol and ethylene glycol, but still could not produce the smaller vesicles from lecithin dissolved in 100% of these polyols (graph at bottom right of Fig. 1). Thus, there was an optimum glycerol content of ~ 40% (w/w) for maximal NLV production. We also observed a small population around 700 nm, most probably aggregates of the NLVs. Thus, it was possible to induce the formation of ~ 100 nm liposomal vesicles by gentle mixing using a magnetic stirrer in a beaker at room temperature.

One could argue that even gentle mixing constitutes actual energy input, which would result in size-reduction of MLVs to NLVs. This would, at least theoretically, preclude calling these NLVs “spontaneous” or “thermodynamically stable”. We thus decided to try to form these NLVs from MLVs without any mixing. A sample of 5% (w/w) Sunlec25 lecithin was dissolved in 100% glycerol until a clear dispersion was achieved. We then diluted this stock to 50% (w/w) with water without any stirring at all. Figure 2 shows the results of this dilution without any mixing. Both the glycerol and ethylene glycol samples partially transformed to NLVs, while the ones prepared in propylene glycol did not. While we proved the point that it is possible to form NLVs spontaneously, we did not explore why propylene glycol did not allow for NLV formation.

Phapal and coworkers^29^ carried out a very elegant and detailed study on the formation of spontaneous liposomes using single phosphatidylcholine molecules in an ethanol–water system. They designed an experiment and apparatus where phosphatidylcholine molecules dissolved in ethanol would come in contact with a water phase. As the water and ethanol exchanged by diffusion, in the absence of convective mixing, liposomes would spontaneously form. The intrinsic diameter of the liposomes formed in the ethanol–water system agreed with calculations based on Helfrich’s theory^30^. This provided support to the conjecture that a natural length scale dictated by thermodynamics “codes” for a liposome’s size. Of interest in this study is that the intrinsic size of the phosphatidylcholine liposomes was dependent on that nature of the fatty acid chains present. Dimyristoylphosphatidycholine yielded ~ 500 nm liposomes, while dioleylphosphatidylcholine yielded ~ 200 nm liposomes, in the range of what we found. At first, it would seem these results contradict Lasic’s conjecture about the need for phospholipid mixtures to generate some curvature in the phospholipid(s) present so as to decrease the elastic bending energy, as discussed in the introduction. Phapal and coworkers^29^ used one molecule at a time to make spontaneous liposomes. However, ethanol partitions into phospholipid bilayers, causing structural changes, while it also affects the hydration of the phospholipid headgroups^31^. These researchers showed that ethanol partitions in the head group region of the bilayers, with 1.6 ethanol molecules per lipid molecule in the gel phase and 1.2 ethanol molecules per lipid molecule in the fluid phase. This partitioning into the headgroup region would increase the effective area of the polar headgroups, thus decreasing the CPP. A decrease in CPP increases the effective curvature of the phospholipid bilayer, favoring vesicle formation. An increase in curvature leads to a decrease in the bending modulus (see Eq. (1)), which would then enhance the spontaneous formation of liposomes. This is not a binary phospholipid-water system, but rather a ternary phospholipid-water–ethanol system.

**Fig. 3:**
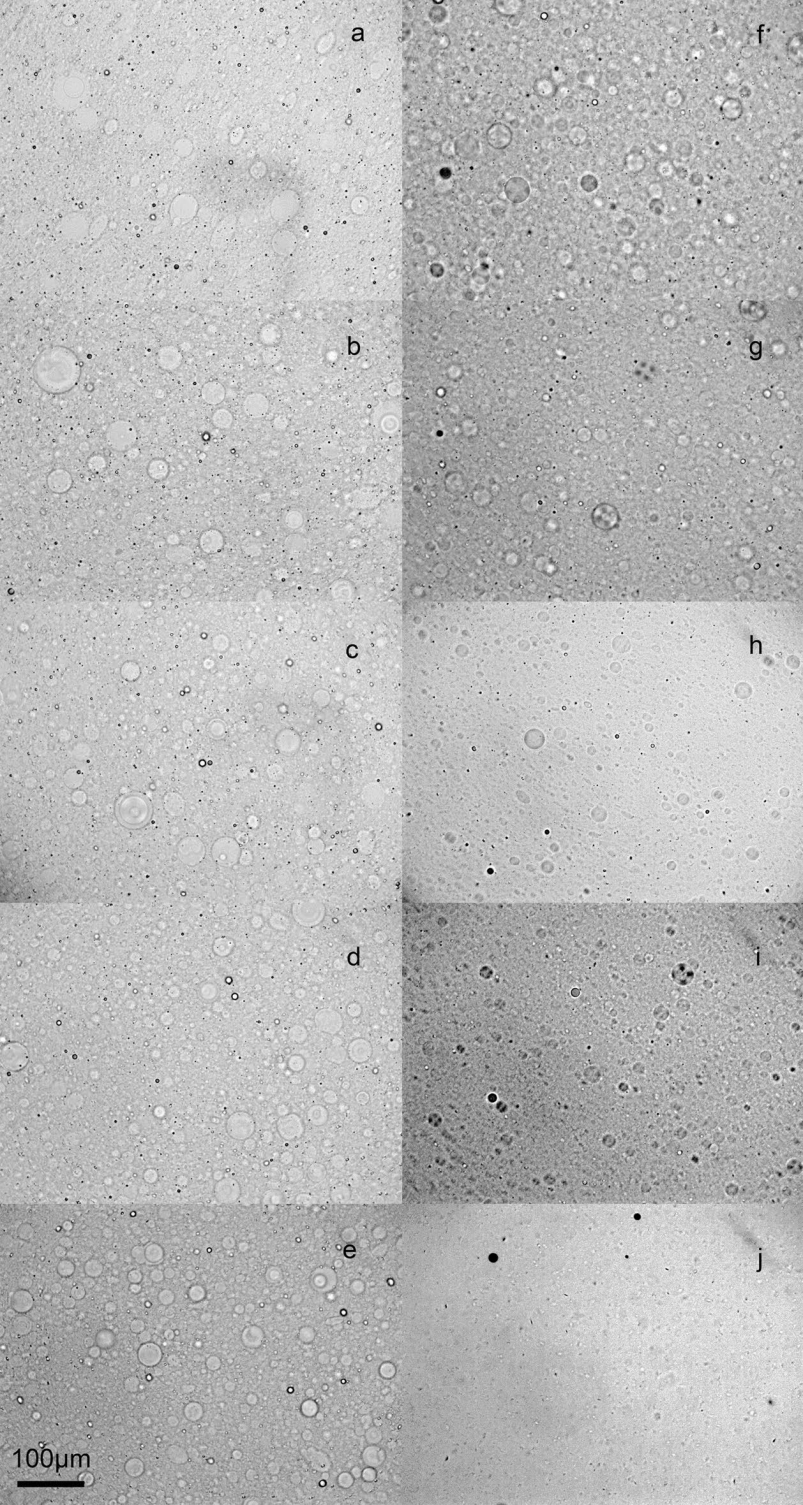
Brightfield light micrographs of 5% (w/w) liposomal preparations of Sunlec25 lecithin in different water-glycerol mixtures, 0% (w/w) glycerol (**a**), 10% (w/w) glycerol (**b**), 20% (w/w) glycerol (**c**), 30% (w/w) glycerol (**d**), 40% (w/w) glycerol (**e**), 50% (w/w) glycerol (**f**), 60% (w/w) glycerol (**g**), 70% (w/w) glycerol (**h**), 80% (w/w) glycerol (**i**), and 100% (w/w) glycerol (**j**).

**Fig. 4:**
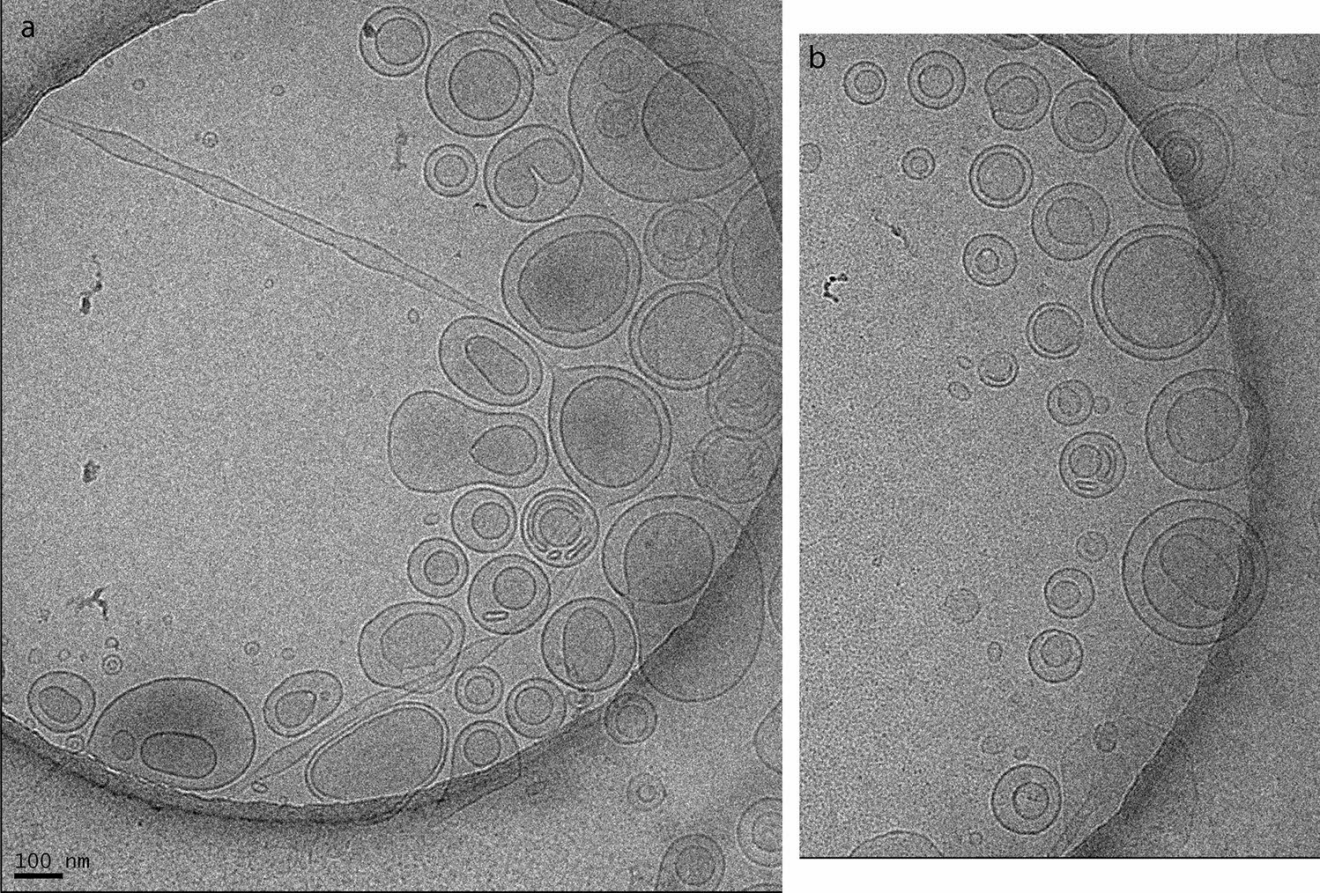
Cryogenic transmission electron micrographs of 10% liposomal suspensions of Sunlec25 lecithin in 50% (w/w) glycerol in deionized water.

Figure 3 shows brightfield micrographs of the different 5% (w/w) Sunlec25 liposomal preparations at different glycerol contents. This figure shows the polydispersity in the preparation and the fact that no MLV liposomes were evident at 100% glycerol. While larger structures are present even with 100% glycerol (Fig. 3, panel j and Fig. 1, 100% glycerol), they are not MLVs, as in the samples containing water. The presence of these non-MLV larger structures in 100% glycerol is consistent with light scattering data. Figure 4 is a cryogenic TEM of 10% Sunlec25 lecithin in 50% glycerol. The micrograph suggests that many of the liposomes are the multivesicular type, but mostly di-vesicular. However, the variability in the images obtained precludes the precise identification of the type of smaller vesicles produced.

**Fig. 5:**
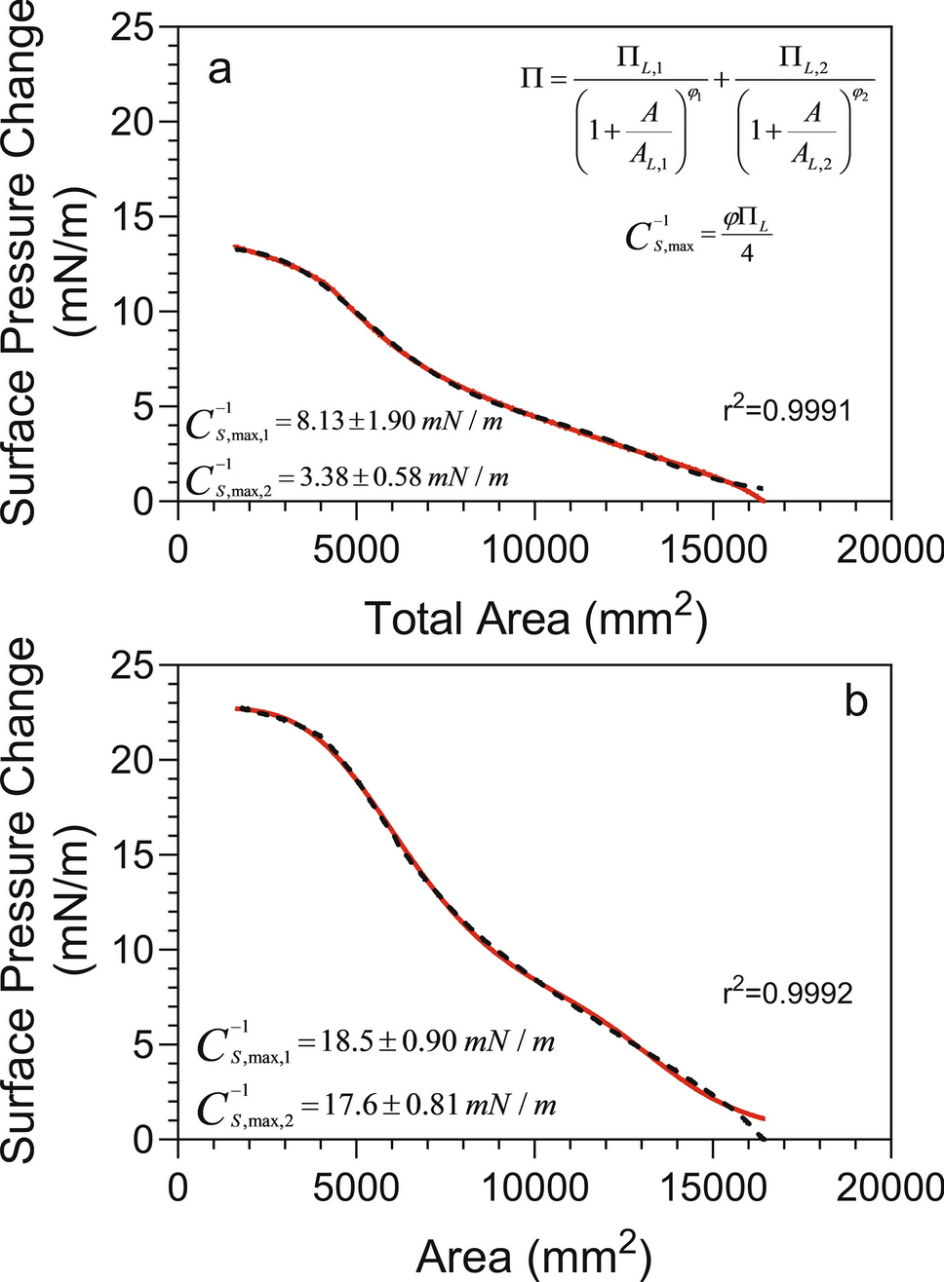
Compression isotherms of 0.01% liposomal dispersions of Sunlec25 lecithin liposomal preparations. (**a**) Liposomes prepared and compressed in 62% (w/w) glycerol, (**b**) Liposomes prepared and compressed in deionized water.

The ease with which MLVs broke down to smaller vesicles in the presence of glycerol, suggests a possible weakening of the bilayers, which upon application of a mild external stress upon mixing, broke down into smaller structures. Langmuir trough surface compression results corroborated this hypothesis. Figure 5 shows the surface compression curves for 5% (w/w) Sunlec25 liposomes in 62% (w/w) glycerol (panel *a*), and in water (panel *b*). The curves were best modelled as having two different compression regions, each with its own compression modulus. This could, for example, represent compression of liposomes sitting at the air–water surface to their 2D close-packed state, followed by deformation of their membranes as the surface is further compressed. Based on this conjecture, the compression modulus of the liposomes decreased from 18.5mN/m in water to 8.13 mM/m in the presence of 62% (w/w) glycerol. This large decrease in the modulus is indicative of a weakening of the membrane, which would make it more sensitive to external momentum transfer fields. Although we have stable liposomes in the liquid phase, we propose that when phospholipid bilayer vesicles migrate to the water–air surface, the outer leaflet of the bilayer unfolds onto the surface, to create a monolayer. The inside monolayer surrounding the water core would then remain as such at the surface, on top of the newly created monolayer, as proposed in a previous study^32^. This contrasts with the classic deposition of a phospholipid monolayer from an organic solvent used in these types of studies. Thus, the surface pressures measured in our experiments should have a strong contribution from the strength of the walls of the vesicles being compressed along with the monolayer.

Using X-ray diffraction, McDaniel and coworkers^33^ reported a swelling of egg PC membranes, as well as dipalmitoylphosphatidylcholine (DPPC) in the presence of glycerol. They reported a maximum swelling of egg PC at ~ 0.1 mol fraction glycerol, which corresponds to 36% mass fraction. Egg PC swelled from 63 to 72 Å, and then shrunk down. For DPPC, the maximum was at ~ 0.25 mol fraction glycerol, which caused swelling from 64 to 72 Å. They attributed this effect to changes in the dielectric permittivity of the fluid space, which weakens van der Waals attractive interactions between bilayers^34^. Thus, glycerol swelled membranes by weakening the attraction between bilayers. This could be the mechanism behind the observed decrease in compressional moduli of liposomes in the presence of glycerol at a mass fraction of 62%, which corresponds to a mol fraction of 0.24, and is in the range of maximum effects observed. Thus, glycerol decreases van der Waals forces between bilayers, causing them to swell and become softer.

This effect can also be understood using the formalism of the Lifshitz approximation to the Hamaker coefficient in van der Waals’ interactions^35^. Gögelein and coworkers^36^ and Gräbner and coworkers^37^ explained the phenomenon based on the fact that the dielectric constant of glycerol is much lower than that of water, while its refractive index is higher. When the absolute values of these parameters are introduced into the Lifshitz expression, one can calculate a net decrease in the magnitude of the Hamaker coefficient, which translates to a decrease in attractive interactions between macromolecular structures^36^. The result of this would be a weakening of bilayer-bilayer interactions within a multilamellar structure, which then would be mechanically weaker and thus able to be broken down by relatively low external shear stresses, until a thermodynamically stable size is reached. This size would be dictated by the intrinsic curvature of the ensemble of phospholipids present.

McDaniel and coworkers^33^ also reported that the bilayer spacing became progressively smaller as the glycerol concentration was increased. They reported a massive drop in the range 0.4 to 0.5 mol fraction glycerol, which corresponds to ~ 80% (w/w) glycerol. In this region they even observed interdigitation between the long fatty acid chains in the bilayer. These chains were being “shoved” into each other. The reader should keep in mind this result from McDaniel and coworkers^33^, since it can help explain the next set of results.

**Fig. 6:**
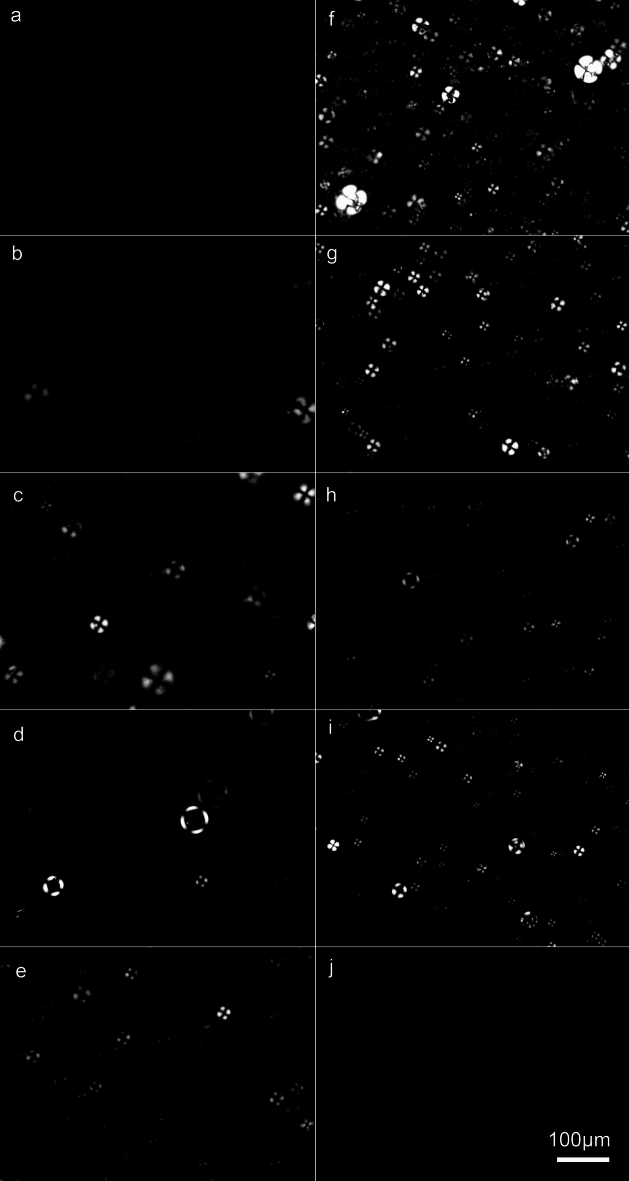
Polarized light micrographs of 5% (w/w) liposomal preparations of Sunlec25 lecithin in different water-glycerol mixtures, 0% (w/w) glycerol (**a**), 10% (w/w) glycerol (**b**), 20% (w/w) glycerol (**c**), 30% (w/w) glycerol (**d**), 40% (w/w) glycerol (**e**), 50% (w/w) glycerol (**f**), 60% (w/w) glycerol (**g**), 70% (w/w) glycerol (**h**), 80% (w/w) glycerol (**i**), and 100% (w/w) glycerol (**j**).

Figure 6 shows polarized light micrographs of the liposomes in progressively higher glycerol concentrations. Interestingly, the liposomes in water and 10% glycerol displayed very weak birefringence. Between 20 and 30% (w/w) glycerol, the liposomes’ birefringence increased drastically.

Birefringence is the optical property of a material having a refractive index that depends on the polarization and propagation direction of light. Optically anisotropic materials are said to be birefringent. The key here is that isotropic materials do not display birefringence. That is observed in our case for liposomes in water. Birefringence can be induced by stress and is referred to as “stress birefringence”^38,39^. What we are observing here is the consequence of nanoscale stress on the phospholipid bilayers due to weakening of van der Waal’s attraction forces and the hydration of the polar headgroups. Birefringence is not observed when the phospholipids are in 100% glycerol. This result confirms that while aggregates exist even without water, their structure is markedly different compared to when water is present. It is evident that water is needed for vesicle formation.

**Fig. 7:**
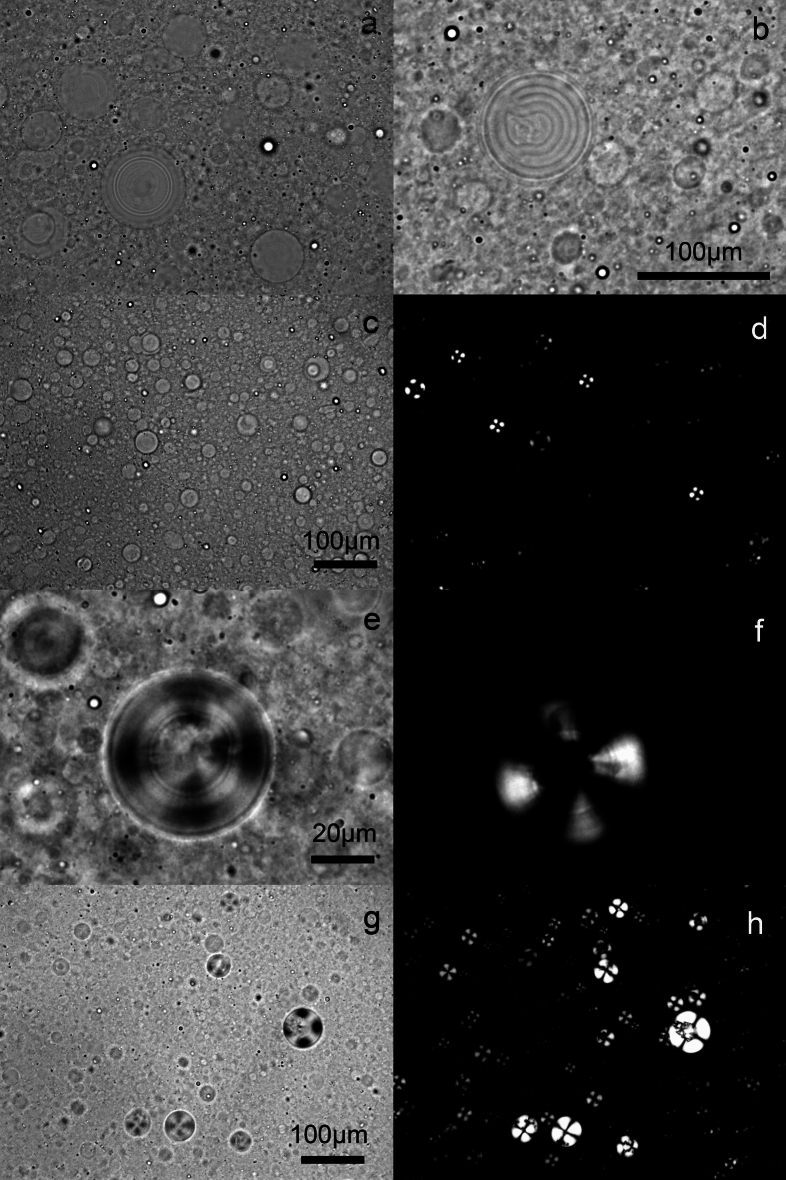
Brightfield and polarized light micrographs of different 5% (w/w) Sunlec25 lecithin liposome preparations. (**a**) 10% (w/w) glycerol, (**b**) 10% (w/w) glycerol, (**c**) and (**d**) corresponding brightfield and polarized light micrographs of 40% (w/w) glycerol liposomes, (**e**) and (**f**) 50% (w/w) glycerol, (**g**) and (**h**) 62% (w/w) glycerol.

Figure 7 shows a variety of images of the liposomal preparations. Figure 7a and b show multilamellar liposomes of Sunlec25 sunflower lecithin in 10% (w/w) glycerol. At this low glycerol concentration, a significant number of the lecithin liposomes are still the MLV type. There isn’t enough glycerol present to decrease intermolecular forces between bilayers and weaken them, thus allowing the conversion of MLVs to NLVs.

In the samples that follow the exact same image of a liposomal preparation is shown under both brightfield conditions and under crossed polarizers. It is interesting to note that not all liposomes display birefringence; on average, the larger liposomes do. It is possible that only the MLV liposomes display stress birefringence. Figure 7c-d, e-f, and g-h correspond to liposomes in 40%, 50% and 62% (w/w) glycerol, respectively. From a practical perspective, polarized light microscopy could serve as a quality control tool to judge whether a treatment is causing stress birefringence, which in turn can mean that the structures have been weakened and could be structurally modified.

**Fig. 8:**
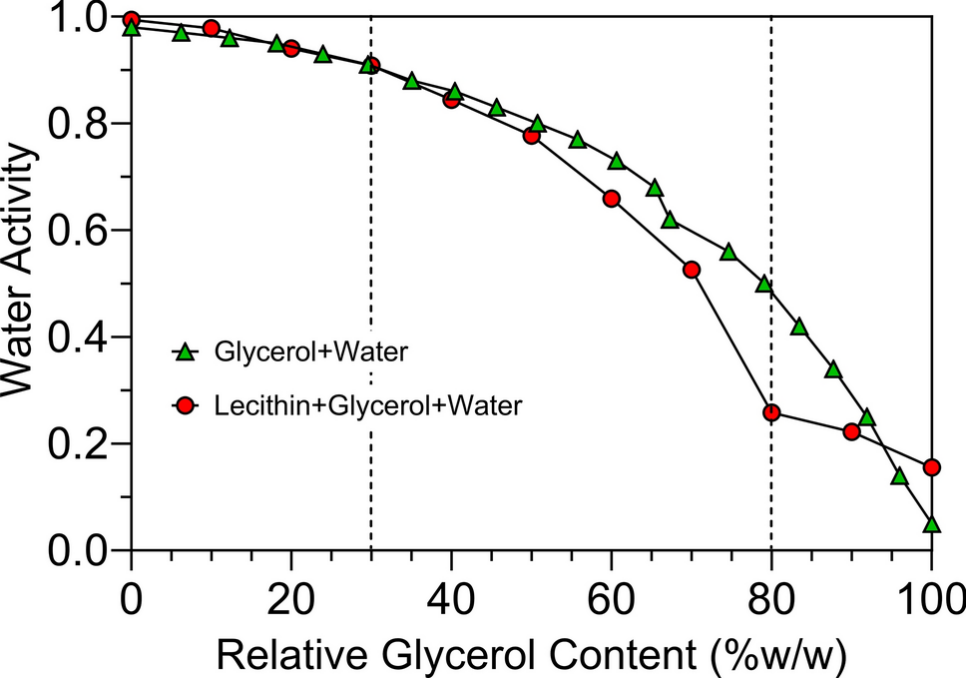
Water activity as a function of glycerol content (w/w%) in for 5% Sunlec25 lecithin liposomes (red symbols). For comparison, data from Nakagama and Oyama^40^ for glycerol-water mixtures was adapted and plotted here (green symbols).

Figure 8 shows the dependence of the water activity of these lecithin-water-glycerol solutions on glycerol content. Here we show the behavior of just glycerol-water mixtures (green symbols), as well as 5% (w/w) Sunlec25 liposomes in water-glycerol mixtures (red symbols).

**Fig. 9:**
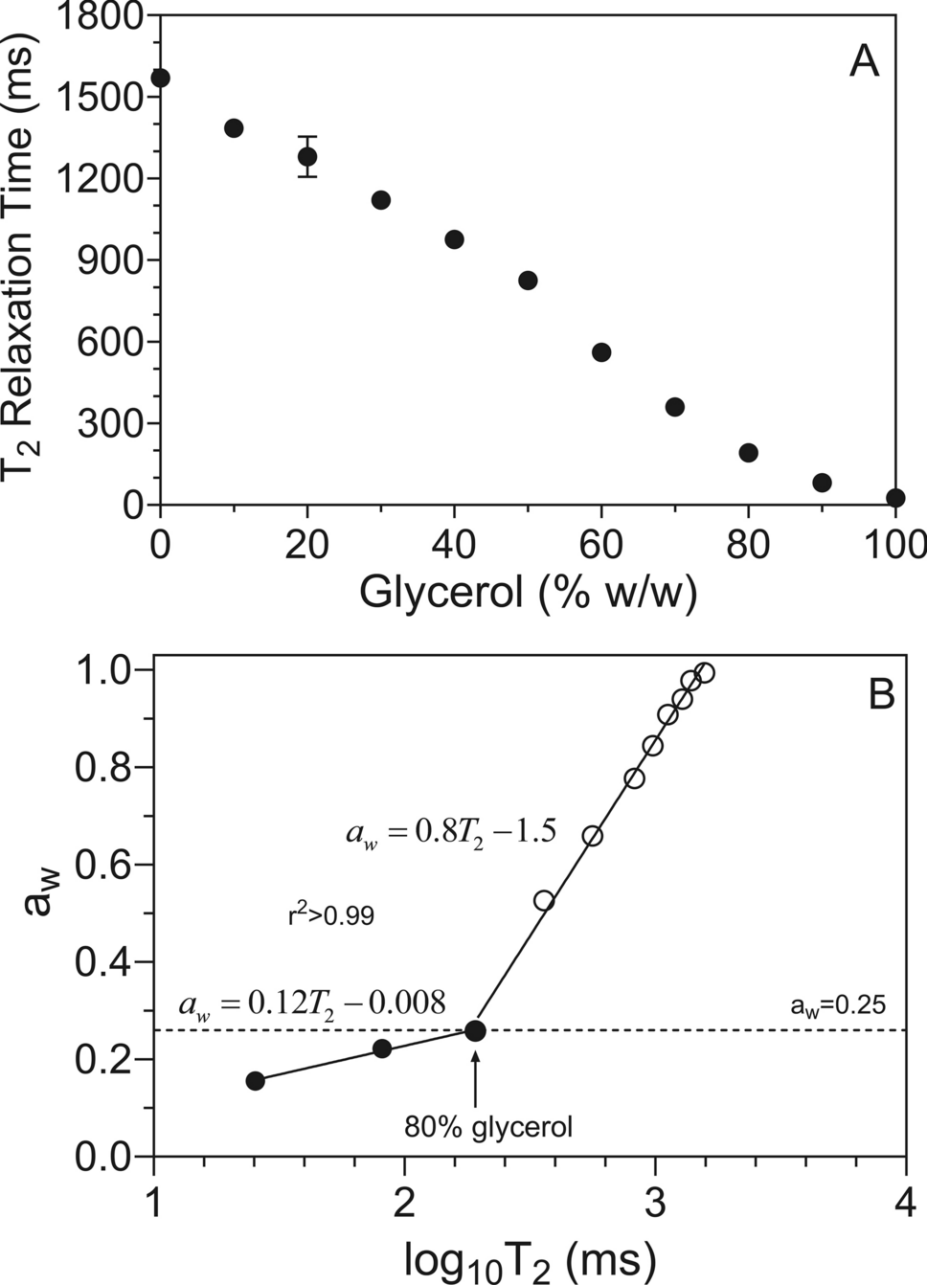
(**a**) Changes in the proton spin-spin relaxation time (T_2_) as a fuction of the glycerol-water mixtures containing 5% (w/W Sunlec lecithin 25 liposomes. (**b**) Experimentally-determined correlation between the water activity of water-glycerol solutions containing 5% (w/w) Sunlec25 lecithin liposomes, and the corresponding T_2_.

The behavior is not the same. In the absence of liposomes, the changes in water activity are gradual, while the liposome-containing solutions displayed a pronounced break at 80% (w/w) glycerol. Interestingly, this is the exact point where McDaniel and coworkers^33^ saw a large drop in the bilayer thickness of DPPC liposomes. This is also the point where the stress-birefringence weakened in our samples. On the other hand, the isotherms started diverging above 30% (w/w) glycerol, which is the concentration where the first stress-birefringence was observed. Glycerol is known to alter the hydrogen-bonded structure of water and to replace water on the surface of membranes^34^. Nakagawa and Oyama^40^ also reported changes in the hydrogen bonded structure of water in this region. It is possible that these changes in hydrogen bonding in the medium cause stress within the bilayers, which allows for their reorganization with minimal energy input. We also measured the spin–spin relaxation time (T_2_) of water in the liposomal suspensions at different glycerol concentrations (Fig. 9a). We then established a quantitative relationship between T_2_ and the water activity (Fig. 9b), and the break at 80% glycerol became more prominent. This figure brings to light the concept that the mobility of water molecules below a water activity of 0.25 is highly restricted since this is a region of monolayer coverage of water on surfaces and supramolecular structures.

**Fig. 10:**
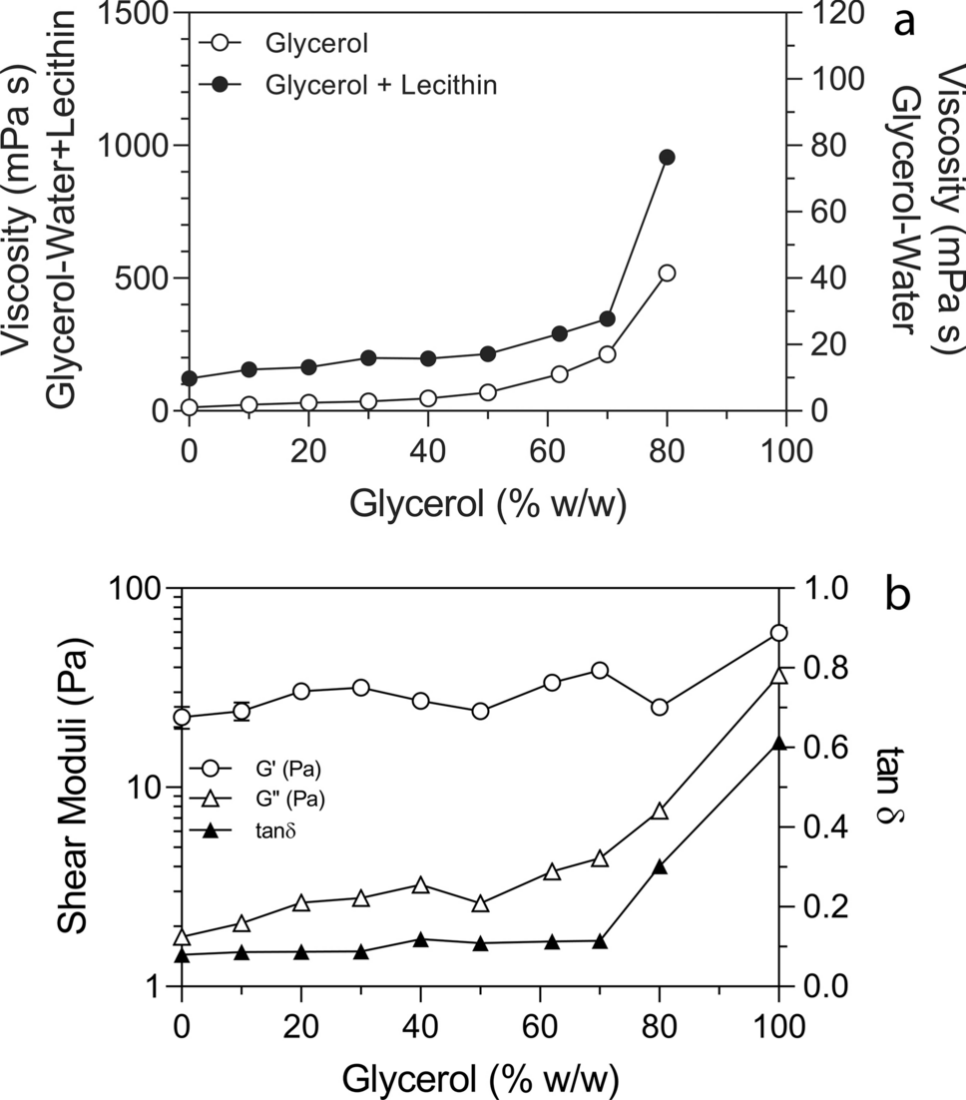
(**a**) Dynamic viscosity changes as a function of glycerol content (w/w %) for water-glycerol solutions with and without 5% Sunlec25 lecithin liposomes. (**b**) Small-angle oscillatory shear moduli for the water-glycerol solutions containing 5% (w/w) Sunlec25 lecithin liposomes. G’ = storage modulus, G” = loss modulus, tanδ = G”/G’.

Figure 10a shows how the viscosity of the liposomes in glycerol-water mixtures changes as a function of glycerol content, while Fig. 10b shows the behavior of the small-deformation oscillatory shear dynamic moduli for the same solutions. The viscosity of the liposomal suspension, as well as the glycerol-water solutions increased gradually to about 70% (w/w) glycerol. Above this, viscosity increased exponentially. The liposome suspensions in glycerol-water solutions always had a higher viscosity than the glycerol-water mixtures, and the break at 70% was more abrupt. Changes in the dynamic moduli of the liposomal suspensions in glycerol-water are shown in Fig. 10b. The storage modulus, G’ and loss modulus, G” increase equally as function of glycerol content, and thus the tanδ (G”/G’) remains constant to 70% glycerol. Above this, there is an abrupt and large increase in the tanδ. This increase indicates a significant transition in the viscoelastic properties of the liposomal dispersion in glycerol from elastic to viscous behavior, *i.e.*, the liposomes lose solid-like behavior and become more liquid-like. This is probably related to the decrease in van der Waals forces between bilayers/liposomes, which results in a decrease in the inter-liposomal interactions, with concomitant structure loss.

## Conclusions

To conclude, in this study we have shown that it is possible to make nanosized liposomal vesicles from multilamellar vesicles prepared in aqueous mixtures using crude, deoiled sunflower lecithin, without the need for high pressure homogenization or membrane filters. The formation of thermodynamically stable vesicles is also aided by having a multicomponent mixture of phospholipids, which yields an effective non-zero intrinsic curvature in the molecular mix. This decreases the bending elastic energy of the vesicles, making them more sensitive to external stresses. In addition, glycerol addition weakens van der Waals’ attractions between bilayers within MLVs, weakening the liposomal wall. The stressed liposomal wall is more sensitive to external stresses and can fall apart more easily. Moreover, glycerol replaces water in the polar headgroups of the phospholipids and decreases their critical packing parameter, also inducing the formation of liposomal vesicles. The spontaneous creation of NLVs from crude, deoiled lecithin upon glycerol addition represents a practical and economical way to scale-up liposomal manufacture for the food and cosmetic industries, thus making liposomal encapsulation of bioactives less expensive and easier to implement.

## Supplementary material

Theoretical formalism used in the calculation of the Critical Packing Parameter of different phospholipid structures reported in Table 2 of this work.

## Supplementary Information

Supplementary Information.

## Data Availability

All data is available by request to the corresponding author.
