# Supplementary material for: Spontaneous nanosized liposome formation from crude dried lecithin upon addition of glycerol
Source: Sci Rep. 2024 Dec 2;14:29931. doi: 10.1038/s41598-024-80970-6 (PMC11611919; doi:10.1038/s41598-024-80970-6)
Supplement: Supplementary file 1 — Supplementary Information. [file 41598_2024_80970_MOESM1_ESM.pdf]

Notes on the Development of a Critical Packing Parameter routine.

James A. Quinn, November, 2018, Norgwyn Montgomery Software, Inc.

The Critical packing parameter (CPP) is a theoretical framework for determining the type of aggregation formed by surfactants (i.e. as spherical or cylindrical micelles, or vesicles, or flexible or fixed bilayers.)

I. The model:

The framework used by MMP+ is:

CPP	Aggregation form
<0.35	spherical micelles
0.35-0.4	spherical or cylindrical micelles
0.4-0.55	cylindrical micelles
0.55-0.6	cylindrical micelles, vesicles or flexible bilayers
0.6-0.85	flexible bilayers or vesicles
0.85-0.95	flexible bilayers
0.95-1.15	planar bilayers
>1.15	inverted micelles or material is not a surfactant

The basic model is:

CPP = Hydrophobic volume/(Hydrophobic length*area of the hydropobic/hydrophilic interface)

or CPP = V/(L*A)

(reference 3)

Since the units are angstroms cubed/(angstroms squared*angstroms), CPP is unitless.

In our old model we used the van der Waal's volume of the hydrophobic portion of the molecule (in surfactant, this usually is a hydrocarbon chain.) In the literature, the molar volume (Molecular weight/specific gravity) was used instead, giving larger numbers. To be consistent with the literature, we changed the method of determining V to:

V = 54.6 + 0.124*(T-298) + Number of CH2,CH groups*(26.9 + 0.0146*(T-298)) - 6.7 for benzene ring - 0.75*(=CH carbon)

This is approximately equal to the van der Waal's volume multiplied by 1.67.

Where T is the temperature in degrees Kelvin, and 25 C is the default temperature (Model is modified for benzene and =CH, but otherwise as from reference 1)

In our old model we used the geometrical maximum length for L. A problem arose from calculating lengths of surfactants with more than one hydrophobic chain, e.g. lecithin, where the maximum length would be drawn diagonally from the start of one chain to the end of the other. We substituted a method from the literature:

L = 1.5 + 1.265*(longest contiguous carbon chain)

(Model from reference 1, the 1.5 accounts for the H that is found at the end of the chain in a CH3 group.)

Note that for double chain surfactants, L will be the same length as a single chain surfactant, but will have double the volume and often this results in surfactants that aggregate in bilayers.

The calculation of the interfacial area between the hydrophobic and hydrophilic portion of the surfactant is more difficult to calculate as it depends, not on geometry, but on steric and charge repulsions and interfacial tension of the hydrophobic portion of the molecule and water:

We used the thermodynamic model of Nagarajan (1,2,4) for two terms:

1) A term for the area at the interface between water and the hydrophobic portion of the molecule, which I will refer to as interfacial repulsion (I) where:
   I = interfacial tension/kT * $(a-a_o)$
   Where a_o is the area of the hydrophobe at the interface (V/L) and a is the area covered by the hydrophilic portion of the surfactant. If is less than or equal to a then I=0 (and a is set to a_o if it is larger than a_o and a is set to a_p otherwise. A_p is the area of the area covered by the hydrophilic part of the molecule.)
   K is Boltzmann's constant and T is degrees Kelvin.
   Interfacial tension = s_s _ s_w -2.0*psi*$(s_s*s_w)^{1/2}$
   Psi = 0.55
   S_s=35.0 – 325$M^{-2/3}$ – 0.098*(T-298)
   S_w = 72.0 -0.16*(T-298)
   M = molecular weight of the hydrophobic surfactant tail.

2) A term for the steric interactions of the hydrophilic portion of the molecule is calculated as:

$S = -\ln(1-[a_p/a])$

There are also terms needed to explain charge repulsion terms between the hydrophilic head groups in the micelle, vesicle or lamellae. We determined these terms using multiple regression. The significant factors were dipole moment, distance from the hydrophilic/hydrophobic interface to the nearest formally charged atom, distance from the interface to counter-ions and distance between + and – charge in zwitterionic surfactants. The model follows in the next section.

II. Results of the calculations

CPP = hydrophobic volume/hydrophobic length*interfacial area

Interfacial area is mainly determined by geometry and charge of the hydrophilic portion of the molecule). Since the volume and length terms are easily calculated, the term that must be modeled is interfacial area. We obtained the following model:

Model of Interfacial_Area

Model coefficients and standard errors:

---

parameter	coefficient	standard error	t	probability
intercept =	-5.0067024	21.5276	0.232571	0.817281
Charge_Distance	3.1341326	0.33281	9.41717	1.05987E-11
CounterIonDistance	-126.70697	12.4541	10.1739	0.000000000001172
Dipole_From_CNDO	6.5515051	1.00974	6.48833	0.0000000973612
InterfacialEnergy	1903.5416	205.024	9.28447	1.57112E-11
Hydrophobic_Volume	-0.043693103	0.0105997	4.1221	0.000183752
ZwitterionDistance	-10.401539	1.55719	6.6797	0.0000000525083
Area_Polar (a_p)	10.913517	1.22261	8.92638	4.59436E-11
Number_of_EO	-6.7786021	1.34238	5.0497	0.0000101045
Steric_Repulsion	-147.3772	25.8276	5.7062	0.00000122305
Charge_Distance crossed with Area_Polar				
	-0.12543458	0.0396633	3.16248	0.00298276
Charge_Distance crossed with Steric_Repulsion				
	-14.504686	1.65751	8.75087	7.81784E-11
CounterIonDistance crossed with CounterIonDistance				

	22.895006	2.49472	9.1774	2.16191E-11
CounterIonDistance crossed with Hydrophobic_Length				
	0.21147464	0.0650173	3.25259	0.00232652
Dipole_From_CNDO crossed with Dipole_From_CNDO				
	-0.038471341	0.00935078	4.11424	0.000188194
Dipole_From_CNDO crossed with Area_Polar				
	-0.26491725	0.0309651	8.55535	0.000000000141943
InterfacialEnergy crossed with ZwitterionDistance				
	-137.10551	28.6167	4.79109	0.0000229834
Hydrophobic_Volume crossed with Number_of_EO				
	0.0089605805	0.00114175	7.84815	0.00000000127136
Hydrophobic_Volume crossed with Steric_Repulsion				
	0.19568428	0.0278056	7.03759	0.0000000166268
ZwitterionDistance crossed with Hydrophobic_Length				
	0.2823166	0.0755368	3.73747	0.000580997
Area_Polar crossed with Hydrophobic_Length				
	-0.22726981	0.0264781	8.58333	0.000000000130296

Analysis of variance

Variation source	df	SS	MS	Statistics
Total (uncorrected)	41	142924.697229		F=126.5741938
Mean	1	138614.7146051		rsquare=0.9921614
Total (corrected)	40	4309.9826239		s=1.2996947
Regression	20	4276.198498		213.8099249
Residual	20	33.7841259	1.6892063	

Note: probability of significant F =<0.0001

Printout of response values, predicted values and residuals:

	observed	predicted	residual
distearyl phosphatidylcholine	43.799999	46.222988	-2.4229898
dipalmityl phosphatidylcholine	43.799999	44.836685	-1.0366848
palmityloleylphosphatidylcholine	43.799999	41.339325	2.4606762
di-trans-oleylphosphatidylethanolamine	60.400002	59.529564	0.8704344
di-cis-olyeylphosphatidylethanolamine	59.299999	58.966843	0.33315602
di-16-ethylstearylphosphatidylethanolamine	59.400002	58.709961	0.6900391
di-16-methylstearylphosphatidylethanolamine	56.099998	58.512978	-2.4129791
di-17-methylstearylphosphatidylethanolamine	60.099998	59.239143	0.86085743
di-17,17-dimethylstearylphosphatidylethanolamine	59	58.421909	0.57809204
Didodecyltrimethylammonium chloride	60.790001	61.312916	-0.52291405
dodecyltrimethylammonium chloride	56	55.515312	0.48468888
dihexadecylphosphate	45.130001	45.136818	-0.0068162745
octoxynol-10	47.200001	47.353405	-0.15340361
decyl maltoside	57	56.786678	0.21332105
laureth-8	50.060001	50.01318	0.046819463
laureth-12	60.099998	61.676334	-1.5763342
laureth-16	68.690002	66.507454	2.1825445
laureth-20	72.360001	72.819565	-0.45956242
laureth-32	70.800003	70.9795	-0.17950107

ceteth-8	48.52	49.003876	-0.48387423
ceteth-12	55.509998	54.561806	0.94819337
ceteth-16	63.599998	63.147827	0.45217183
ceteth-20	72.269997	73.262749	-0.9927488
ceteth-32	80.177002	80.025856	0.15114647
distearyl phosphatidic acid	52	51.862854	0.13714665
distearyl phosphatidyl inositol	66.779999	66.765327	0.01467095
distearyl phosphatidylglycerol	76.919998	77.081429	-0.16142601
dioleylphosphatidylserine	50.82	50.468395	0.35160527
C16 sphingomyelin (N+(CH3)3)	46.150002	45.882042	0.26795846
C16 sphingomyelin (N+H3)	42.209999	42.608261	-0.39826173
sodium hexadecyl sulfate	71.599998	71.625427	-0.025429966
sodium myristyl sulfate	69.5	69.476089	0.02391251
sodium lauryl sulfate	67.400002	67.363693	0.036306705
sodium decyl sulfate	65.400002	65.365349	0.034647372
sodium octyl sulfate	63.5	63.530796	-0.030797362
N-octylbetaine	60.599998	60.802513	-0.20251487
N-decyl betaine	58	57.797359	0.2026412
laurylbetaine	56.48	56.083988	0.39601356
myristylbetaine	55.560001	54.961105	0.59889531
cetylbetaine	53.119999	54.152447	-1.0324451
lauryl glucamine	34	34.237255	-0.23725539

Probability cut-off: 0.05

This model contains more terms (20) than it should for the limited number of observations (40). It will be improved as we receive new data points.

III. References:

1. A. Nagarajan and E. Ruckenstein (1991): "Theory of Surfactant Self-Assembly: A Predictive Molecular Thermodyanmics approach", Langmuir 1991, 7, 2934-2969. We obtained the formulas for V, L and interfacial tension from this paper. There are many facets in the elaborate theory presented in this paper for which we are making empirically-based short-cuts, notably in the calculation of charge repulsions and attractions.

2. R. Nagarajan (2001): "Molecular Packing Parameter and Surfactant Self-Assembly: The Neglected Role of the Surfactant Tail", Langmuir 2002, 18, 31-38. Our results for SDS and similar molecules is based on Table 2. Our models for aggregation number (g) is also lifted from this paper (Table 1) as well as formulas for micellar V and A (also Table 1).

3. J. Israelachvili, D.J. Mitchell, B.W. Ninham (1976) J. Chem. Soc. Faraday Trans. 2, 1976, 72, 1525. The original frequently cited presentation of the packing parameter.

4. Ramanathan Nagarajan, 2007, "Theory of Micelle Formation Quantitative Approach to Predicting Micellar Properties from Surfactant Molecular Structure." In book: Structure-Performance Relationships in Surfactants, Edition: 2nd, Chapter: 1, Publisher: Marcel Dekker, Editors: K. Esumi and M. Ueno, pp.1-110. The model for copolymers of propylene oxide and ethylene oxide is derived from this chapter in a book.

5. Paul E. Harper, David A. Mannock, Ruthven N. A. H. Lewis, Ronald N. McElhaney, and Sol M. Grune (2001), “X-Ray Diffraction Structures of Some Phosphatidylethanolamine Lamellar and Inverted Hexagonal Phase”, Biophysical Journal, Volume 81. November 2001, pps 2693–2706. This reference provided the values for the phosphatidylethanolamine compounds.
